# Supplementary material for: The Zinc Finger Transcription Factor BbCmr1 Regulates Conidium Maturation in Beauveria bassiana
Source: Microbiol Spectr. 2022 Feb 9;10(1):e02066-21. doi: 10.1128/spectrum.02066-21 (PMC8826823; doi:10.1128/spectrum.02066-21)
Supplement: SUPPLEMENTAL FILE 1 — Supplemental material. Download SPECTRUM02066-21_Supp_1_seq15.pdf, PDF file, 0.6 MB [file spectrum02066-21_supp_1_seq15.pdf]

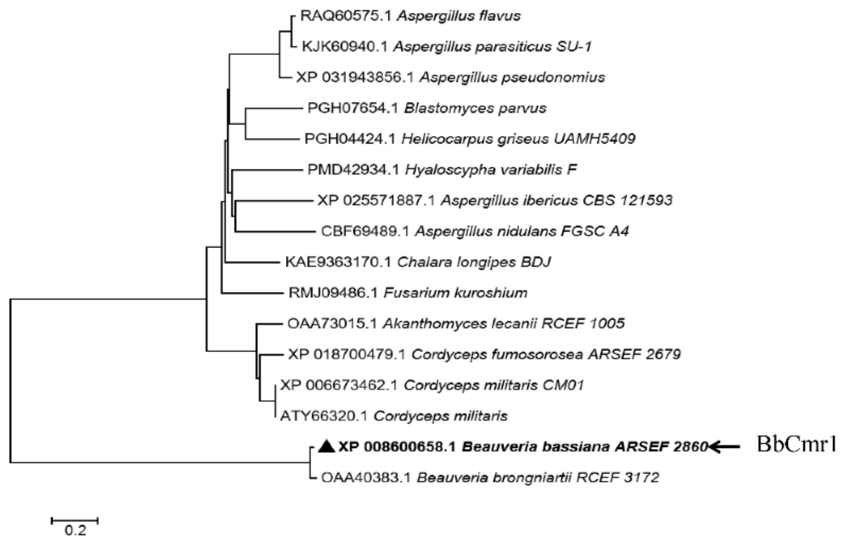

FIG S1 Phylogenetic analysis of BbCmr1.

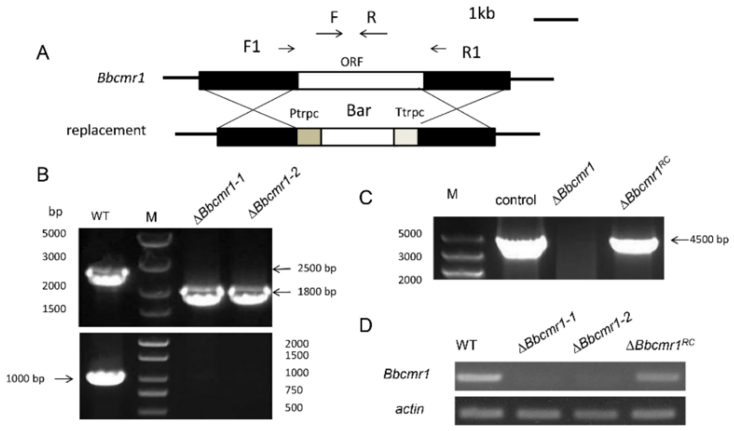

FIG S2 Screening of the *Bbcmr1* deletion strain and the complementation strain. (A) Schematic diagram of the *Bbcmr1* deletion mutant (*ΔBbcmr1*). (B) PCR screening of *ΔBbcmr1* deletion strains. In the upper panel, primers located upstream of the left arm and downstream of the right arm were used. In the bottom panel, primer pairs located in the deletion region of *Bbcmr1* were used, and no fragment was observed in the mutant strain. (C) The complementation strain (*ΔBbcmr1*^{RC}) was confirmed by PCR. (D) *ΔBbcmr1* and *ΔBbcmr1* ^{RC} were confirmed by RT–PCR, and the *actin* gene was used as a reference.

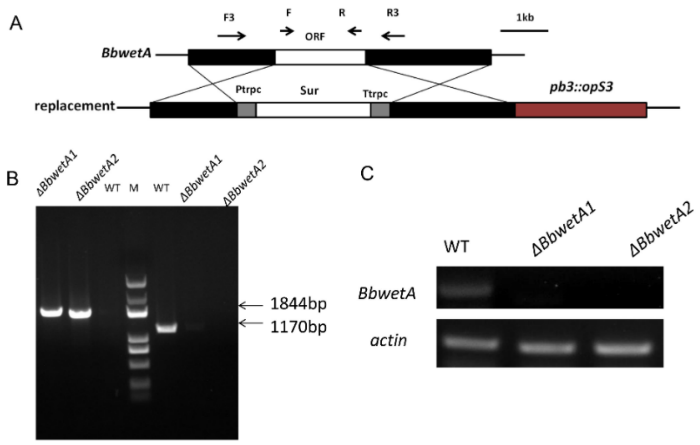

FIG S3 Screening of the *BbwetA* deletion strain. (A) Schematic diagram of the *BbwetA* deletion mutant (*ΔBbwetA*). (B) PCR screening of *ΔBbcmr1* deletion strains. In the left plane, primers located upstream of the left arm and in the Sur cassette were used. In the right plane, primer pairs located in the deletion region of *Bbcmr1* were used, and no fragment was observed in the mutant strain. (C) *ΔBbwetA* was confirmed by RT–PCR, and the *actin* gene was used as a reference.

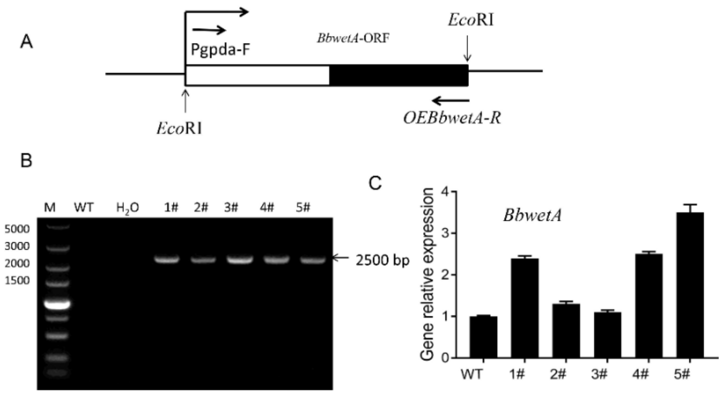

FIG S4 Screening of *BbwetA* overexpression in *Bbcmr1* deletion strains. (A) Schematic diagram of overexpression of *BbwetA* in the deletion mutant (*ΔBbcmr1*). (B) Primers were used to detect the *pgpda* and *BbwetA* genes. (C) Relative transcript levels of *BbwetA* in potential *ΔBbcmr1/BwetA*^{OE}.

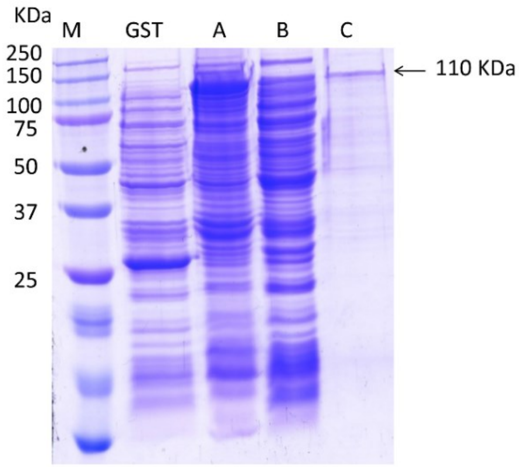

FIG S5 SDS–PAGE assay of BbCmr1 expressed in *E. coli.* GST, *E. coli*-produced GST protein. A and B, cell lysate precipitate and supernatant. C, purified BbCmrl protein. M, protein marker.

Table S1 Primers in this study

**For RT-PCR**	
qbrlA-F/R	GACCAGTTCAACAGACAAG//CAGTAATCTTCGTGCTTCTC
qabaA-F/R	GCAAGTCTCCAGCCATAT//CTCCTCTTCGTCATAGTAGTC

qwetA-F/R	CGCAGACGAATTTGACTT//GCTGGTGGTTGAATACAT
qcmr1-F/R	ATGACGTCAATGCGCAGGTA//GGGACGAAATGTACCGGTGT
qBBA_06297-F/R	TACTTACTCTCAGGGCGGCT//CAAAAGCGGGGAGGTTGTTG
qBBA_01877-F/R	CCTCGTCTTTGAGTCGCAGT//AGTTCTGATTCTGGCCGACG
qBBA_03717-F/R	TTGCCGATGTCCGTATCGAG//TTCCCTCCGTGTTGTGGATG
qBBA_05929-F/R	TATACCAAGACCCCCAGCGA//AGCGATGGGGAAACCTTGAG
qBBA_09760-F/R	CTTTTTGTGTACGCCGGCAA//ATGTCTGTGCTGGCGTTGTA
qBBA_05808-F/R	CACACCAAGCCTGAGTCTGT//TTACAGAGCAAGGACAGCGG
qBBA_07526-F/R	AGCTCTCCCCTCTACACTGG//TAGCTGTACTGGCACTGCAC
qBBA_00246-F/R	CGCCTCTAGACCCCAGGTAT//ATGTCGCAGTAGCTGGCAAT
qBBA_09129-F/R	GAGGACGATCGAGCTCAGTG//GAGAAGACCGCCCAGATAGC
qBBA_04013-F/R	ATCAAGCGACAAGGTTCCGT//CAAGGGCAGGAGTAGTGGTG
qBBA_00676- F/R	ATGTCACGACGCAGACTGTT//CTCGCACGATAGGCGTAAGT
qBBA_02994- F/R	TGATGCGCTGGCTCGATATT//AGAAGAGAGCGCTACCTGGA
qBBA_02123- F/R	AGGCATCATCTCGCGTGTAG//CCGCTCATGGAGTTTGTCCT
qBBA_08495- F/R	GGAGCGACTACCATCTCGTG//CAGTATGAACGCAATGCGGG
**For deleted gene**	
cmr1-F1	CAGCTATGACCATGATTACGAATTCGCGTGGTGGGAGTCGAGC
cmr1–R1	TCTTCTGTCGACACTAGTGAATTCCGCAGTCTCGCTCTGGGTT
cmr1-F2	TTAGAGGTAATCCTTCTTTCTAGACGCTGGACGGGTCGTTTTGG
cmr1-R2	GCATGCCTGCAGGTCGACTCTAGATGGCACAGATTGAAAAGC
cmr1-F	CGAAGCGTTGAGGATTGCAGAG
cmr1-F1	CAGCTATGACCATGATTACGAATTCTCCCATGCCCACATAAACCT
wetA–R1	TCTTCTGTCGACACTAGTGAATTCATGTAAAACGGCTCGGCGAC
wetA-F2	TTAGAGGTAATCCTTCTTTCTAGAATGTATTCAACCACCAGCGG
wetA-R2	GCATGCCTGCAGGTCGACTCTAGAGGATTCACATGTAGATGGCT
wetA-F	ACCTCAATGTAGCCAAGCCT
**For overexpression gene**	
Pb3-wetA-down	GGACGATGTCCAGAGAGCCATTGTTATTGATTAAAAGGGTGA
OEwetA-F	TCACCCTTTTAATCAATAACAATGGCTCTCTGGACATCGTCC
OEwetA-R	CTGTCGACACTAGTGAATTCTCAAAGCTCAAACCCTTGCTCG
Pb3-cmr1-down	CCTTGACCGTTGTTTCCATTGTTATTGATTAAAAGGGTGAGG
OEcmr1-F	CCTCACCCTTTTAATCAATAACAATGGAAACAACGGTCAAGG
OEcmr1-R	CTGTCGACACTAGTGAATTCCCTTCGTCAGTAATCATCCA
**For promoter (gene) fused with eGFP(RFP)**	
cmr1-pro-F	CAGCTATGACCATGATTACGAATTCGCGTGGTGGGAGTCGAG
cmr1-pro-R	CGCCCTTGCTCACCATGATGACGACGCAAAGGTC
GFP-F	GACCTTTGCGTCGTCATCATGGTGAGCAAGGGCG
GFP-R	TCTTCTGTCGACACTAGTGAATTCTCACTTGTACAGCTCGTCCA
mcherry-F	TTCTGGTGGTGGTTCTGGTGTGAGCAAGGGCGAGGAG
mcherry-R	TCTTCTGTCGACACTAGTGAATTCTTACTTGTACAGCTCGT
rds1-F(RFP)	CAGCTATGACCATGATTACGAATTCTACTGGACAATGGCTTC
rds1-R(RFP)	AACCACCACCAGAACCACCACCAGCCTGATACACACCC
**For yeast one-hybrid assay**	
BrlA -42AD-F	TGCCTCTCCCGAATTCATGCAGTTTGAGTCGTCGAG
BrlA -42AD-R	AGTCCAAAGCTTCTCGAGTCAGTAATCTTCGTGCTTCT
Cmr1 -42AD-F	TGCCTCTCCCGAATTCATGGAAACAACGGTCAAGGC
Cmr1-42AD-R	AGTCCAAAGCTTCTCGAGCCTTCGTCAGTAATCATCCA
WetA-P1-F	ATTGAAAAGCTTGAATTCCTGCATCCCACCAACCAATC
WetA -P1-R	GAGCACATGCCTCGAGGAGACGGGCCTCACGCTTTT
WetA -P2-F	ATTGAAAAGCTTGAATTCAAAAGCGTGAGGCCCGTCTC
WetA -P2-R	GAGCACATGCCTCGAGAAACCAGAGAGCTTCAGCCG
WetA -P3-F	ATTGAAAAGCTTGAATTCACAATCACGGCTGAAGCTCT

WetA -P3-R	GAGCACATGCCTCGAGGGCCCTGAGGATGTATGATT
placzi-brlA-F	AATTCACCTGACCTGACAAGTTGACAAGTAGAAAAAAAAAAAGGAAA AAAAAAAAAGAAAAGGAACC
placzi-brlA-R	TCGAGGTTCCTTTTCTTTTTTTTTTTTCCTTTTTTTTTTCTACTTGTCAA CTTGTCAGGTCAGGTG
placzi-cmr1-F	AATTCCGCGTCGGAATTACGATATTACCCCTAGAGACAATTTTTATTA TAGCTGCC
placzi-cmr1-R	TCGAGGCAGCTATAATAAAAATTGTCTCTAGGGGTAATATCGTAATT CCGACGCGG
$(A4GA3)_3$–F	AATTCAAAAGAAAAAAAGAAAAAAAGAAAC
$(A4GA3)_3$–R	TCGAGTTTCTTTTTTTCTTTTTTTCTTTTG
$(A4G2A2)_3$–F	AATTCAAAAGGAAAAAAGGAAAAAAGGAAC
$(A4G2A2)_3$–R	TCGAGTTCCTTTTTTCCTTTTTTCCTTTTG
**For EMSA assays**	
BrlA-6P-F	CTGGGATCCCCGGAATTCATGCAGTTTGAGTCGTCGAG
BrlA-6P-R	ACGATGCGGCCGCTCGAGTCAGTAATCTTCGTGCTTCT
Cmr1-6P-F	CTGGGATCCCCGGAATTCATGGAAACAACGGTCAAGGC
Cmr1-6P-R	ACGATGCGGCCGCTCGAGCCTTCGTCAGTAATCATCCA
brlA-EMSA-F1	GGAAAAAAAAAAAAGAAAAGGAACCCACAACTCAAGTCT
brlA-EMSA-R1	AGACTTGAGTTGTGGGTTCCTTTTCTTTTTTTTTTTTCC
cmr1- EMSA-F1	CGGAATTACGATATTACCCCTAGAGACAATTTTTATTATA
cmr1- EMSA-F1	TATAATAAAAATTGTCTCTAGGGGTAATATCGTAATTCCG
